# Comparative Effect of Allicin and Alcoholic Garlic Extract on the Morphology and Infectivity of *Eimeria tenella* Oocysts in Chickens

**DOI:** 10.3390/ani12223185

**Published:** 2022-11-17

**Authors:** Salwa Mahmoud Abd-ELrahman, Sara Abdel-Aal Mohamed, Samar Elsayed Mohamed, Manal F. El-Khadragy, Ahmed Kamal Dyab, Nashwa Hamad, Marwa M. Safwat, Asmaa A. E. Nasr, Abdulsalam A. M. Alkhaldi, Ahmed Gareh, Ehab Kotb Elmahallawy

**Affiliations:** 1 Department of Parasitology, Faculty of Veterinary Medicine, Assiut University, Assiut 71515, Egypt; 2 Private Veterinary Clinic, Assiut University, Assiut 71515, Egypt; 3 Department of Biology, College of Science, Princess Nourah bint Abdulrahman University, P.O. Box 84428, Riyadh 11671, Saudi Arabia; 4 Department of Parasitology, Faculty of Medicine, Assiut University, Assiut 71515, Egypt; 5 Department of Pathology and Clinical Pathology, Faculty of Veterinary Medicine, Assiut University, Assiut 71515, Egypt; 6 Department of Avian and Rabbit Diseases, Faculty of Veterinary Medicine, Assiut University, Assiut 71515, Egypt; 7 Department of Poultry Diseases, Animal Health Research Institute, Assiut 71515, Egypt; 8 Biology Department, College of Science, Jouf University, Sakaka 72388, Saudi Arabia; 9 Department of Parasitology, Faculty of Veterinary Medicine, Aswan University, Aswan 24101, Egypt; 10 Department of Zoonoses, Faculty of Veterinary Medicine, Sohag University, Sohag 82524, Egypt

**Keywords:** viability, infectivity, oocyst shedding, disinfectant

## Abstract

**Simple Summary:**

Avian coccidiosis is one of the major diseases threatening the poultry industry in Egypt and worldwide. As such, developing a new oocysticidal to control this disease seems crucial. The present study examined the in vitro effects of allicin and alcoholic garlic extract at different concentrations as natural disinfectant candidates compared with KOH 5% as a chemical disinfectant against *Eimeria tenella* oocysts and the in vivo infectivity of these treated oocysts in chickens. The morphological changes in the treated oocysts were examined using light microscopy and scanning electron microscopy. The in vivo infectivity was evaluated using a gross and histopathological examination of the cecal tissues to determine the most significant changes. The study showed promising results for using Allicin at a concentration of 180 mg/mL and garlic at a concentration of 360 mg/mL in reducing the number of oocysts, the sporulation percentage, and the number of oocysts with marked deformities. The sporulated oocysts exposed to Allicin and alcoholic garlic extract revealed the lowest infectivity and pathogenicity, resulting in a minor gross lesions score. In addition, restoration of the major reported histopathological changes compared to oocysts sporulated in media containing the KOH 5%. Collectively, this study provides novel information regarding the potential use of natural extracts of allicin and alcoholic garlic as environmentally eco-friendly disinfectants in poultry farms to reduce the spread of coccidiosis.

**Abstract:**

Avian coccidiosis remains one of the major parasitic diseases that threaten the global poultry industry. Since prevention is superior to treatment, this study focuses on eliminating the infection outside the host. To determine their effect on the viability of *Eimeria tenella* oocysts in vitro, allicin and alcoholic garlic extract, which are natural, less toxic, and inexpensive products, were compared to KOH 5% (chemical disinfectant) using an in vitro culture system. Three concentrations of allicin (45, 90, and 180 mg/mL) and alcoholic garlic extract (90, 180, and 360 mg/mL, were used. Subsequently, destructive and sporulation-inhibiting effects on *Eimeria* oocysts were detected using light and electron microscopy. Young chickens were infected with treated sporulated oocysts to determine their effect on infectivity. After 7 days pi, the percentage of excreted oocysts (oocyst shedding) was determined, and the chickens were slaughtered for histopathological examination of the cecal tissues. Under an electron microscope, allicin at a concentration of 180 mg/mL and alcoholic garlic extract at a concentration of 360 mg/mL demonstrate a high oocysticidal activity with severe destruction of the oocyst wall and the appearance of pores. In addition, both concentrations directly affected the infectivity of sporulated oocysts by reducing the shedding of oocysts and the pathological lesions of infected young chickens. We concluded that the ability of Allicin and alcoholic garlic extract to eliminate *Eimeria* oocysts makes them superior to chemical disinfectants as a disinfectant.

## 1. Introduction

Coccidiosis in chickens is among the most significant parasitic diseases affecting the poultry industry. The disease is associated with significant production losses, high morbidity, and mortality rates in excess of 50%, resulting in enormous economic losses [1]. The disease is caused by the genus *Eimeria* [2], a large genus with over 1800 species infecting different hosts, involving mammals and birds [3]. There are seven *Eimeria* species that infect chickens. *Eimeria tenella* is one of the most destructive, causing fulminating lesions primarily in broilers. Only *E. tenella* inhabits the ceca of chickens exclusively, whereas the asexual life cycle of *E. necatrix* occurs in a different portion of the chicken intestine. The infectious stage of *Eimeria* is sporulated oocyst, which is transmitted through the consumption of contaminated food, water, and litter. In addition, the conditions under which most chickens and other birds are raised facilitate the disease’s spread [4].

*E. tenella*-infected chickens exhibit bloody droppings, anemia, emaciation, depression, delayed growth, and low production. Ceca exhibits distention, hemorrhagic spots on the wall, inflammation, necrotic patches, and core formation. Microscopically, noticeable cecal hemorrhage, mucosal necrosis, and significant infiltration of eosinophils and mononuclear cells can be observed [5]. Additionally, *E. tenella* in cecal tissue is characterized by a high oocyst density and clusters of large secondary-stage meronts arranged in the cecal mucosa and submucosa [6,7].

Most chemotherapeutic anticoccidials, such as sulphonamides, amprolium, nicarbazin, halofuginone, and toltrazuril, are still used to control outbreaks of poultry coccidiosis. Different chemical anticoccidial combinations and concentrations effectively control chicken coccidiosis [8]. These drugs mainly target certain developmental stages of the parasite [4]. However, excessive use of chemotherapeutic drugs inevitably results in the emergence of drug resistance among *Eimeria* species, as well as meat residues and consumer health risks [1]. Preventing the infectious stage (sporulated oocysts) and reducing the cost of chemotherapy depends on thorough pre-stocking disinfection [9]. The chemical nature of the eimerian oocyst wall renders it resistant to universal disinfectants and limits the number of disinfectants that can be used. Numerous studies have demonstrated that most disinfectants are ineffective against sporulated *Eimeria* oocysts, despite their destructive effect on non-sporulated oocysts [10,11].

Therefore, the search for alternative, harmless, biologically and environmentally friendly natural anticoccidial agents has become a global imperative [2]. Recently, commercially available, plant-based products have become available for use as anticoccidial feed additives in poultry farming [12,13].

*Allium sativum* (garlic) is one of the most widely utilized medicinal plants [14]. Numerous studies have ascertained the competitive antimicrobial and immunological activity of garlic and its thiosulfinate compounds. (ACSO, SAC, S-Allyl-L-cysteine sulfoxide) is the major biologically active component of freshly crushed garlic extracts. It is readily permeable through artificial and biological phospholipid membranes [15]. Allicin, a reactive sulfur species (RSS), can oxidize cellular thiols, e.g., cysteine residues in proteins [16,17].

Reviewing the available literature, a small number of studies assessed the potential effects of Allicin and garlic extract on the infectivity of *Eimeria* oocyst by detecting in vivo pathogenicity and oocyst shedding. Hence, the present study estimated the in vitro oocysticidal effect of Allicin and alcoholic extracts of *Allium sativum* (garlic) as natural disinfectants to control poultry coccidiosis, and the in vivo infectivity of these treated sporulated oocysts.

## 2. Materials and Methods

### 2.1. Materials 

#### 2.1.1. Allicin and KOH 5%

Allicin oil (Centa Kind Pharmaceuticals, RC# 7797, Nefertari, Limited Co., El-Fayoum, Egypt) was dissolved in 10% dimethyl sulfoxide (DMSO) + 15 μL tween 80 at concentrations of 45, 90, and 180 mg/mL. Furthermore, a KOH 5% solution (El-Gumhya Co., Assiut, Egypt) was made by dissolving 5 g of KOH crystals in 100 mL of normal saline.

#### 2.1.2. Alcoholic Garlic Extraction

Approximately 500 g of peeled *Allium sativum* bulbs were obtained from an Egyptian commercial market and dehydrated at room temperature (30–35 °C) in a dark location for 1 month. The peeled bulbs were then ground in a rotary mortar and left to dry further. In a maceration jar, 50 g of plant powder was thoroughly shaken with 200 mL of 95% ethanol. To prevent alcohol from evaporating, the jar was tightly sealed and stored at room temperature (30–35 °C) for 24 h while shaking. The extract was then filtered through No. 1 Whatman^®^ filter paper. The solvent was extracted using a Rota evaporator at 50–55 °C, followed by air-drying. The yield of dry extracts was calculated and used immediately. 90, 180, and 360 mg/mL concentrations of garlic were dissolved in 10% DMSO + 15 μL tween 80.

#### 2.1.3. Eimeria Oocysts

Cecal *Eimeria* oocysts were collected from naturally infected 30–day-old broiler chickens in the Egyptian Sohag Governorate. Cecal tissue specimens from these birds were fixed in 10% neutral buffered formalin and submitted to histopathological processing and examination [18] to identify *Eimeria* sp. [6,7]. Oocysts were harvested, concentrated, and clarified using a saturated sodium chloride solution before being washed and counted using the McMaster method [19]. Oocysts were allowed to sporulate [20] before being morphologically identified as *E. tenella* [21]. 5 × 10^3^ sporulated oocysts per bird were orally administered to 5-week-old chickens to propagate the sporulation of oocysts. On the seventh day following gavage, the cecal contents of these birds were collected and treated with 5% sodium hypochlorite (4:1) for 25 min to sterilize the non-sporulated oocyst surface and dissolve the debris. The sodium hypochlorite was eliminated by washing three times with 0.9% normal saline and centrifuging at 1500 rpm for 5 min. To avoid any physical disruption, oocysts were handled gently and suspended in normal saline [22]. Subsequently, using the McMaster counting chamber, the number of sporulated oocysts per mL of suspension was adjusted to 6.3 × 10^3^/mL [19].

### 2.2. Experimental Design

#### 2.2.1. Ethical Approval

The Assiut University Faculty of Veterinary Medicine Research Ethical Committee and Review Board approved the study with the approval number aun vet/3/0001. During the in vivo trial, the experimental animals (birds) were handled in accordance with local laws, ethical practices, and regulations, as well as the principles and guidelines for poultry welfare.

#### 2.2.2. Evaluation of the In Vitro Effect of Different Allicin and Alcoholic Garlic Extract Concentrations on the Number and Sporulation Dynamics of *E. tenella* Oocysts

Non-sporulated *E. tenella* 6.3 × 10^3^ oocysts/mL were exposed to three concentrations of allicin (45, 90, and 180 mg/mL), alcoholic garlic extract (90, 180, and 360 mg/mL), and KOH 5% in 6-well tissue-culture plates with a total volume of 3 mL per well. Each concentration was duplicated three times, and the entire experiment was repeated three times. Plates were covered with perforated foil and incubated at 28 °C, 60–80% humidity, and 20 rpm shaking continuously for 48 h (Heidolph Duamax1030 platform shaker). Similarly, untreated control oocysts in 2.5% K_2_Cr_2_O_7_ and untreated oocysts in 10% DMSO + 15 μL Tween 80 were allowed to sporulate under the aforementioned conditions. After 24 and 48 h, the experimental set was examined to track sporulation development and morphological alterations of the oocyst. After 48 h, oocysts from each treatment were washed three times with 0.9% saline, stored at 4 °C, and then counted using the McMaster technique [21]. The oocyst count reduction percentage (48 h post incubation) was calculated using the mean number of oocysts for each treatment [11] according to the equation:

$$\mathrm{Oocysts}\;\mathrm{reduction}\;\%\;=\frac{\mathrm{Mean}\;\mathrm{oocyst}\;\mathrm{No}.\;\mathrm{of}\;\mathrm{control}-\mathrm{Mean}\;\mathrm{oocysts}\;\mathrm{No}.\;\mathrm{of}\;\mathrm{treatment}\;}{\mathrm{Mean}\;\mathrm{oocyst}\;\mathrm{No}.\;\mathrm{of}\;\mathrm{control}}\times 100$$

Based on sporulation (S%) and sporulation inhibition (SI%) percentages, the effect of allicin and alcoholic garlic extract on sporulation dynamics was analyzed. The sporulated oocysts were counted [23], and the inhibitory sporulation percentage was calculated from the equations as suggested by Cedric et al. (2018);

$$\mathrm{Sporulation}\;\mathrm{inhibition}\;\left(\mathrm{SI}\right)\;\%\;=\frac{\mathrm{Sporulation}\;\%\;\mathrm{of}\;\mathrm{control}-\mathrm{Sporulation}\;\%\;\mathrm{of}\;\mathrm{treatment}\;}{\;\mathrm{Sporulation}\;\%\;\mathrm{of}\;\mathrm{control}}\times 100$$

#### 2.2.3. Evaluating the Effect of Allicin and Alcoholic Garlic Extract on the Morphology of *E. tenella* Oocysts 

The morphological changes in the oocysts (sporulated or non-sporulated) induced by allicin, garlic extract, and KOH 5% were evaluated using light microscopy. In addition, a scanning electron microscopy (SEM) examination was performed after fixing oocysts from each treatment in 2.5% fresh glutaraldehyde solution using SEM (Jeol Jsm-54001v) [24].

#### 2.2.4. In Vivo Infectivity of *E. tenella* Sporulated Oocysts Treated with Allicin and Alcoholic Garlic Extract

To determine the infectivity of the treated oocysts, sporulated *E. tenella* oocysts from a prior in vitro treatment were used (Allicin, Alcoholic garlic extract, and KOH), then were washed thoroughly with normal saline and ether (9:3). Then, fifty-three-week-old coccidia-free chickens were divided into five groups (10/each): Non-infected control, infected with untreated oocysts (sporulated in K_2_Cr_2_O_7_), infected with allicin-treated oocysts, infected with alcoholic garlic-treated oocysts, and infected with KOH-treated oocysts. Each infected bird was intra-crop inoculated with 1 × 10^4^ of the respective treatment [25]. Birds had access to food and water until the conclusion of the experiment. On the seventh day following infection, birds from all groups were slaughtered for cecal lesion scoring, oocyst output, and histopathological examination [26].

##### Oocyst Shedding 

Oocysts/gram of cecal contents (OPG) were counted using the McMaster method on each bird slaughtered for gross lesion scoring [27]. The mean OPG of each group was evaluated, and the oocyst’s shedding reduction percentage was calculated:

$$\mathrm{Shedding}\;\mathrm{Reduction}\;\%=\frac{\mathrm{Shedding}\;\mathrm{of}\;\mathrm{control}\;\mathrm{challenged}-\;\mathrm{Shedding}\;\mathrm{of}\;\mathrm{medicated}}{\mathrm{Shedding}\;\mathrm{of}\;\mathrm{control}\;\mathrm{challenged}}\times 100$$

##### Cecal Lesion Scoring

The severity of the gross cecal lesions [28] was rated from 0 to 4 in 5 birds per group, with 0 denoting no lesions and 4 denoting the most severity. The percentage of reduction in lesion severity (RLS %) was estimated from the mean lesion score (MLS) for each group using the equation:

$$\mathrm{RLS}\;\%=100-\left(\frac{\mathrm{MLS}\;\mathrm{of}\;\mathrm{medicated}\;}{\mathrm{MLS}\;\mathrm{of}\;\mathrm{challenged}\;}\times 100\right)$$

##### Histopathological Examination and Scoring

Fresh cecal specimens from five birds per experimental group were formalin-fixed and processed for histopathological analysis. Hematoxylin and eosin were applied to 5 µm-thick paraffin sections and stained with hematoxylin and eosin (H&E). The microscopic examination was conducted by light microscopy (Olympus CX31, Tokyo, Japan) and photographed with a digital camera (Olympus, Camedia C-5060, Tokyo, Japan) in the Photomicrograph Lab of the Department of Pathology and Clinical Pathology, Faculty of Veterinary Medicine, Assiut University [29,30]. All detected microscopic lesions were scored according to their severity, as described elsewhere [11] as follows: 0: normal, 1: minimal severity, 2: mild severity, 3: moderate, 4: marked, and 5: severe.

### 2.3. Statistical Analysis

Statistical Package for Social Science (IBM SPSS for Windows, version 22, Chicago, IL, USA) was used to conduct a one-way analysis of variance (ANOVA) on the data, followed by Tukey’s test as a post hoc test for detecting significance between different treatments. The data were expressed as mean ± SE. It was deemed statistically significant if *p* < 0.05.

## 3. Results

### 3.1. Macroscopic and Microscopic Lesions of Naturally Infected Broiler Chickens

**Figure 1:**
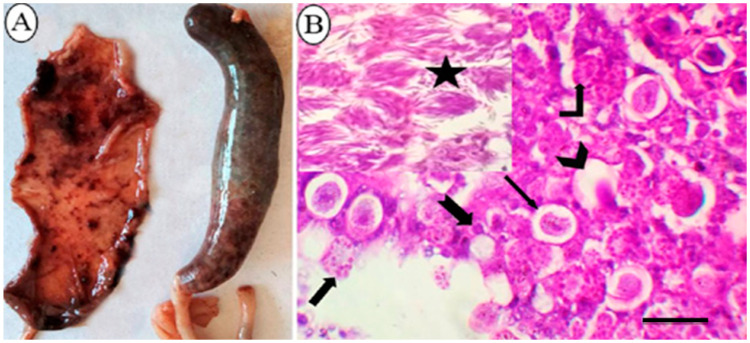
The pathological findings in ceca of *E. tenella* naturally infected broiler chickens. (**A**): Gross appearance of ceca showing severe congestion and dilatation and filled with bloody contents with friable cecal wall and loss of its lining mucosa. (**B**): Histopathological cecal section illustrating the distinct developmental stages of *E. tenella*; mature oocyst with a central nucleus and oocyst wall (Chevron arrow); immature oocyst with a central nucleus and oocyst wall (thin arrow); macrogametes with bordering eosinophilic bodies and a central nucleus (Bent-up arrow); microgametes (Thick arrow); developing schizont (notched arrow), mature second stage schizonts (star) arranged in groups and having several crescent-shaped merozoites (H&E, bar = 50 µm).

Affected chickens had a severely swollen and dilated ceca containing bloody contents, with a friable cecal wall and loss of its lining (Figure 1A). Microscopically, the cecal specimens obtained from suspected cases in the field revealed developmental stages distinguishing *E. tenella* in cecal mucosa (Figure 1B).

### 3.2. In Vitro Effect of Different Concentrations of Allicin and Alcoholic Garlic Extract on the Number and Sporulation Dynamics of E. tenella Oocysts

Data depicted in Table 1 displays the effect of different concentrations of allicin (45, 90, and 180 mg/mL) and alcoholic extract of garlic (90, 180, and 360 mg/mL) compared to the effect of KOH 5% (as a chemical disinfectant) on *E. tenella* oocysts. A highly significant reduction of oocysts number was observed after treatment with allicin at a concentration of 180 mg/mL, followed by an alcoholic garlic extract (360 mg/mL) and allicin 90 mg/mL; 88.3 ± 1.2%, 73.35 ± 2.2%, 72.7 ± 1.7%, respectively, (*p* < 0.001%). KOH 5% reduced oocyst count by 67.4 ± 1.5%. Alcoholic garlic extract (360 mg/mL) significantly lowered sporulation (S) percent (13.6 ± 1.6%), causing the highest sporulation inhibition (SI) percent (73.4 ± 2.8%), followed by allicin (180 mg/mL), 15.9 ± 0.9% S percent, and 68.9 ± 2.1% SI percent. The highest sporulation percent occurred in the KOH 5% treated wells (35.6 ± 1.5%) with an SI percentage of 30.5 ± 2.4%.

### 3.3. Evaluating the Effect of Allicin and Alcoholic Garlic Extract on the Morphology of E. tenella Oocysts 

**Figure 2:**
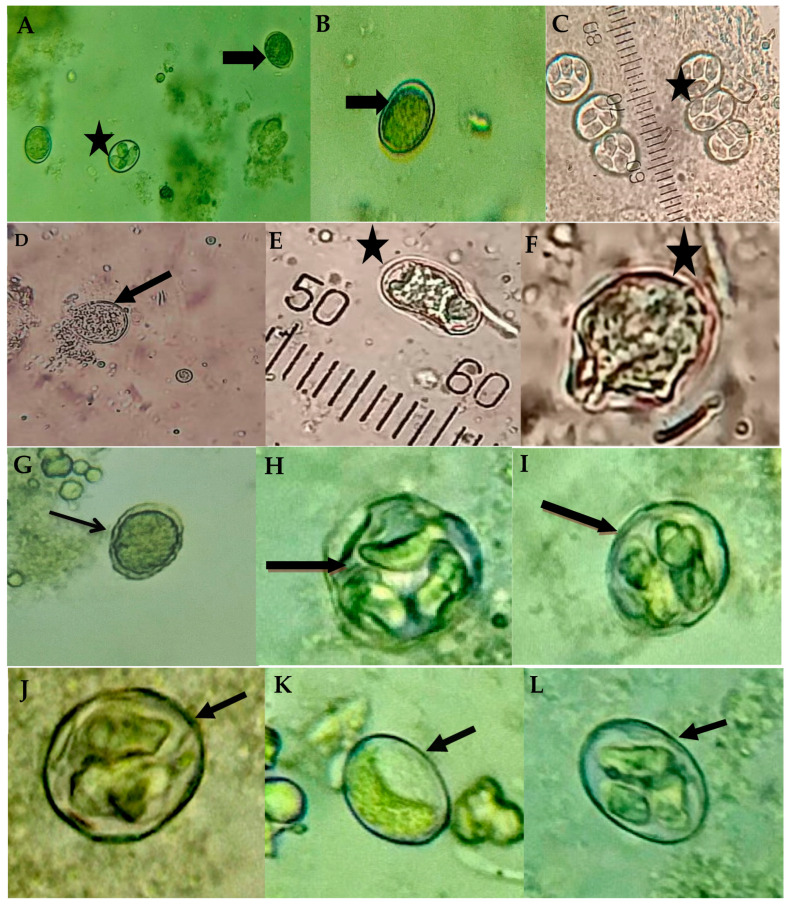
The morphological changes of *Eimeria tenella* oocysts after 24 and 48 h due to in vitro exposure to various treatments. Non-treated control: (**A**,**B**) Non-sporulated (thick black arrow) oocysts with regular, smooth double walls and refractile contents. (**C**) Sporulated tetrasporic dizoic oocysts (black star). Allicin-treated oocysts: (**D**) Non- oocysts were severely deformed with a ruptured wall and leaked contents (thick black arrow). (**E**,**F**) Some oocysts exhibited a shrunken appearance, lost their regular ovoid shape (black star), and contained undifferentiated contents. Alcoholic garlic extract treated oocysts: (**G**) The oocyst wall was corrugated (thin arrow), (**H**,**I**) The contents of the oocyst were disrupted (thick black arrow). Oocysts were treated with 5% KOH; (**J**–**L**) The oocysts’ walls were relatively undisturbed (thick black arrow), but the cyst contents were deformed. Light microscope, 40× and 100×.

The morphological analysis of the treated oocysts by Light microscopy revealed the destructive potential of the allicin and alcoholic garlic extract on *E. tenella* oocysts (Figure 2 and Table 2). The non-treated control oocysts had a typical appearance with smooth walls and refractile contents without deformities. Allicin and garlic extract-treated oocysts depicted several morphological changes, including oocyst wall corrugation and discontinuation with abnormal appearance of the oocysts content, which became opaque and displaced. Leakage of oocyst content (Figure 2B,C) and oocyst deformities were observed. 

**Figure 3:**
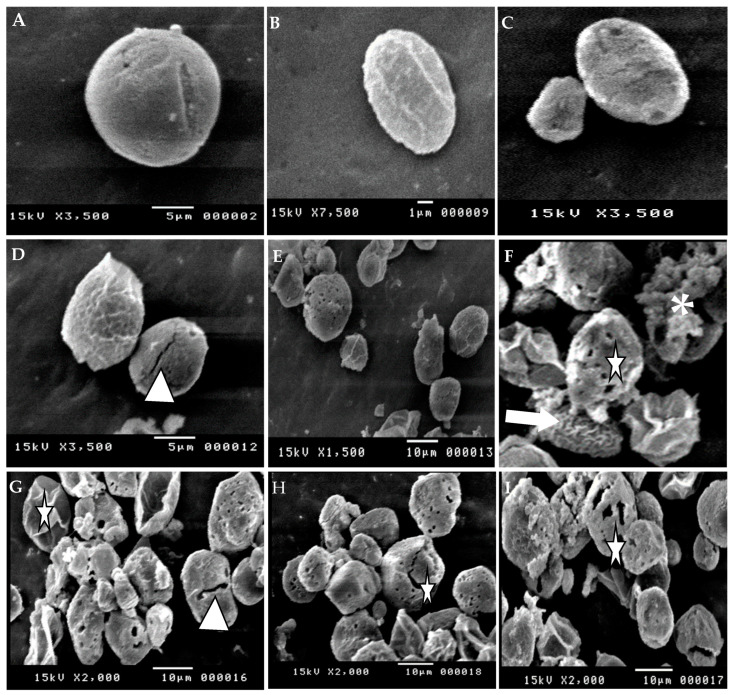
Scanning electron micrograph of *E. tenella* oocysts undergoing sporulation in the presence of allicin and alcoholic garlic extract, 48 h post-incubation. (**A**–**C**) Oocysts in untreated controls were spherical and intact. Allicin and Alcoholic garlic extract-treated oocysts exhibited. (**D**,**E**) Irregular oocysts shape, oocyst walls roughness that became fractured at specific areas (ow, arrowhead). (**F**) Broken non-sporulated oocyst (white arrow) exposing its cytoplasm with distorted amylopectin granules throughout (asterisk). (**G**–**I**) Oocyst lost its wall during early cytokinesis (star). Note the empty oocyst wall (arrowhead) resulting from the destruction of sporocysts and the collapse/shrinking of some oocysts due to the lysis of their contents, ×3500.

In accordance with SEM, the wall of untreated oocysts appeared smooth, intact, and devoid of the micropyle. In contrast, Allicin and alcoholic garlic extracts treated oocysts exhibited marked structural changes as evidenced by an irregular oocyst shape and loss of smoothness, as well as fractures, numerous pits, and pores in the oocyst wall. Some oocysts contracted or shrank as a result of the lysed contents. Also observed was the loss of the external oocyst wall layer, exposing the surface of the underlying layer and its perforations (Figure 3).

### 3.4. In Vivo Infectivity of E. tenella Sporulated Oocysts Treated with the Allicin and Alcoholic Garlic Extract

#### 3.4.1. Reduction of Oocysts Shedding

Infection with allicin-treated oocysts reduced oocyst shedding to 93.7%, followed by garlic extract-treated oocysts, which reduced oocyst shedding to 87.6% (*p* < 0.05). Moreover, oocyst shedding due to infection with KOH 5% treated oocysts was reduced to only 39%. 

#### 3.4.2. Gross Lesion Scoring 

**Figure 4:**
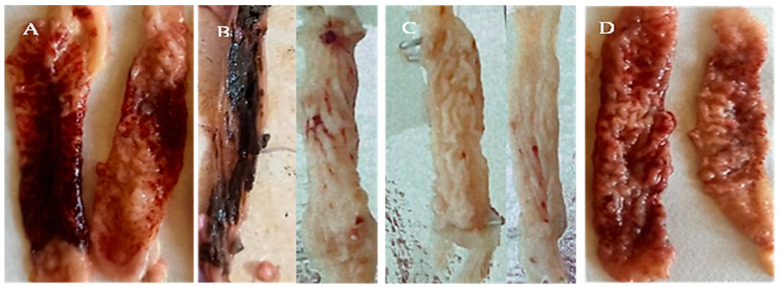
Gross pathological findings in the ceca of E-infected 3-week-old coccidia-free chickens (7 days after infection) *tenella* oocysts sporulated in various solutions. (**A**) Lesion score + 4 was caused by *E. tenella* oocysts that sporulated in 2.5% K_2_Cr_2_O_7_, revealing a sheet of coagulated blood covering the mucosa, complete loss of cecal corrugations, and ulceration. (**B**) Lesion score +1 induced by *E. tenella* oocysts sporulated in allicin (90 mg/mL) solutions; slight thickening of the cecal wall; cecal contents are relatively normal. (**C**) Lesion score +2 was induced by *E. tenella* oocysts that sporulated in an alcoholic garlic extract (180 mg/mL) solution, revealing a moderately thick cecal wall, scattered patechae on the mucosal surface, and tenacious cecal contents. (**D**) Lesion score +3 induced by *E. tenella* oocysts which sporulated in KOH 5%; ceca had a markedly thickened wall, eroded mucosal surface, and bloody cecal contents.

The ceca of birds infected with untreated sporulated oocysts revealed a coagulated blood sheet covering the mucosa, loss of all cecal corrugations, and ulceration (Figure 4A). However, birds infected with allicin-treated oocysts had normal cecal contents and a slight thickening of the cecal wall (Figure 4B). The cecal wall of birds infected with oocysts treated with alcoholic garlic extract was moderately thick, with few patechae dispersed through the mucosal surface and tenacious contents (Figure 4C). Ceca of birds infected with KOH-treated oocysts showed significantly thickened walls, eroded mucosal surfaces, and bloody contents (Figure 4D).

**Figure 5:**
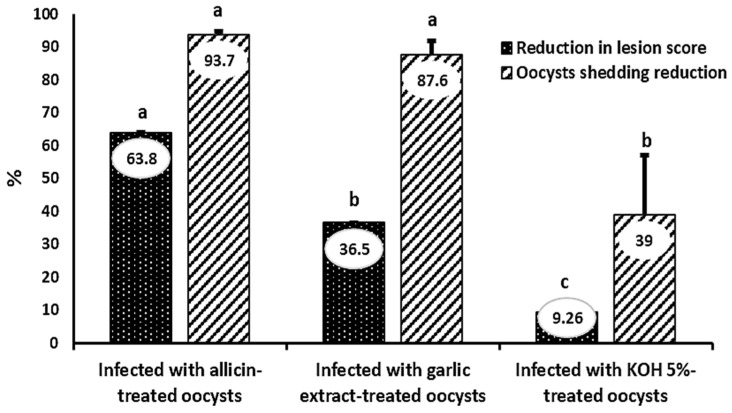
At 7 days post-infection with *E. tenella*, the percentages of reduction in mean lesion scores and oocyst shedding were calculated. Sporulated *E. tenella* oocysts in Allicin, alcoholic garlic extract, and 5% KOH. Significant differences exist between columns with different lettering (*p* < 0.05). Error bars represent mean ± standard errors of the mean.

Allicin-treated oocysts caused the mildest cecal gross lesions expressing the highest RLS % (63.8%), which was significantly higher (*p* < 0.05) than alcoholic garlic extract-treated oocysts causing 36.5% RLS. Cecal damage was significantly greatest in the following infection with oocysts treated with 5% KOH and the lowest RLS% (9.26%) (*p* < 0.01) (Figure 5).

#### 3.4.3. Histopathological Findings and Scoring

**Figure 6:**
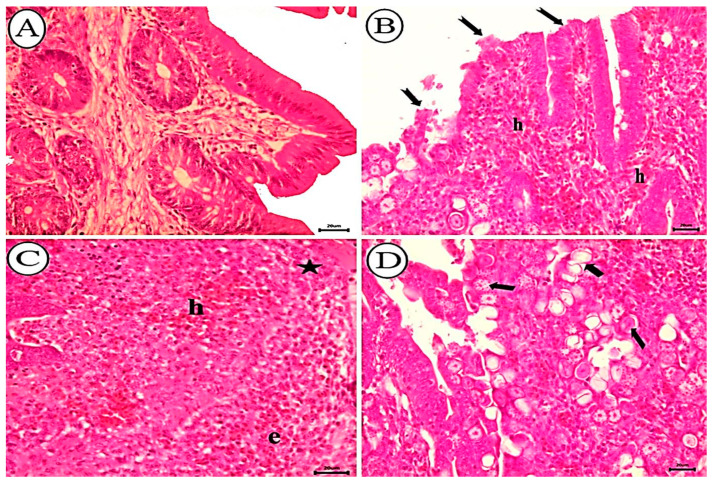
Photomicrograph showing histopathological characteristics of cecal tissues. (**A**) Negative control group of non-infected birds showing normal tissue appearance. (**B**–**D**) Birds infected with non-treated oocysts. (**B**) Significant epithelial shedding (notched arrow) and diffuse mucosal bleeding (h). (**C**) Diffuse mucosal hemorrhages (h), eosinophils (e), and mononuclear cell infiltration (star). (**D**) Various coccidial stages in the villar epithelium and lamina propria (arrow), H&E stain, scale bar = 20 µm.

**Figure 7:**
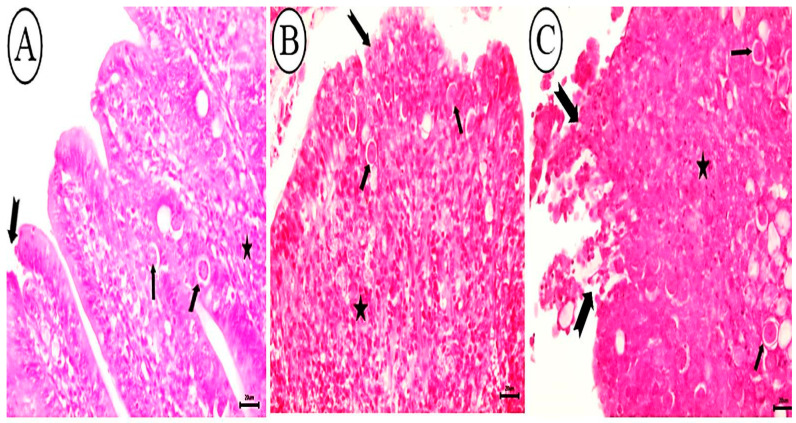
Photomicrograph depicting histopathological characteristics of cecal tissues. (**A**). Birds infected with allicin-treated oocysts exhibited a significant reduction in the number of coccidial stages (arrow), accompanied by slight epithelial necrosis at villar tips (notched arrow) and infiltration of mononuclear cells (star). (**B**) Birds infected with garlic extract-treated oocysts display reduced epithelial necrosis (notched arrow), coccidial stages (arrow), and mononuclear cells in the lamina propria (star). (**C**) Birds infected with KOH-treated oocysts exhibiting villar necrosis (notched arrow), mononuclear cells (star), and parasitic stages in the lamina propria (arrow), H&E stain, bar = 20 µm.

The scoring of histopathological findings in sections of the cecum from different groups is shown in Table 3. Negative control, non-infected birds exhibited normal intestinal epithelial appearance in cecal sections (Figure 6A). However, the ceca of birds infected with untreated sporulated oocysts exhibited severe hemorrhagic inflammatory lesions characterized by massive necrosis, sloughing of villar epithelium, and diffuse mucosal hemorrhages (Figure 6B). Diffuse infiltration of eosinophils and mononuclear cells in the lamina propria and submucosa (Figure 6C) accompanied by the widespread distribution of *E. tenella* in its various stages. Also observed were *E. tenella* in the villar, cryptal epithelium, and lamina propria (Figure 6D). Birds infected with allicin-treated oocysts exhibited a marked decrease in necrotic changes, a slight infiltration of mononuclear cells in the lamina propria, and a significant reduction in the number of coccidial stages in the villar and cryptal epithelium (Figure 7A). Birds infected with oocysts treated with alcoholic garlic extract exhibited a reduction in lamina propria necrotic changes, mononuclear cell infiltration, and parasitic stages (Figure 7B). Ceca of birds infected with KOH-treated oocysts exhibited obvious villar necrosis, diffuse mononuclear cell infiltration, and parasite developmental stages (Figure 7C).

## 4. Discussion

The overuse of anticoccidial medications has resulted in the development of drug resistance in addition to the deposition of their residues in tissues and organs [12,31]. Previous studies have evaluated the effectiveness of various herbs and herbal by-products in preventing the massive losses caused by coccidiosis in the poultry industry [12,13,32]. Allium sativum has many activities, including anti-inflammatory, antimicrobial, antioxidant, antiprotozoal, antifungal, anticancer, and hepatoprotective effects [32,33,34,35]. It has traditional nutritive and medicinal applications as an anti-infective, anti-parasitic, and anthelmintic agent [36]. Moreover, garlic demonstrated effective prophylactic and therapeutic effects against coccidian parasites [37]. Allicin induces changes in the intestinal microbiota of broiler chickens, has an antioxidant effect on *Eimeria* oocysts in broiler chickens [38], and efficiently inhibits the efficient sporulation of *E. tenella* [13]. However, little is known about the potential effects of garlic extract and allicin on the in vivo pathogenicity and oocyst shedding by *E. tenella.* The objective of this study was to evaluate the in vitro and in vivo efficacy of various concentrations of alcoholic extracts of Allium sativum and Allicin as herbal drugs in broiler chickens infected with *E. tenella* compared to KOH 5% (chemical disinfectant).

As shown in Table 1, garlic extracts 360 mg/mL and Allicin 180 mg/mL significantly reduced the number of oocysts by 73.5 and 88.3%, respectively, followed by KOH5% (67.35%). It appears that the reduction of oocyst counts in the feces of treated groups is attributable to bioactive components of Allicin and garlic that can interact with cytoplasmic membranes and alter their cations permeability, resulting in the disruption of vital processes in the parasite cells and, ultimately, their death [39]. In addition, several previous studies indicated that these herbal extracts might stimulate immunity by increasing the antibody response, which directly kills the sporozoites [40,41]. As shown in Table 2, the various concentrations of garlic extract and allicin had a marked effect on oocyst sporulation. According to a review of the available literature, several previous studies employ various aqueous extracts against *E. tenella*, *Eimeria acervulina*, and *Eimeria maxima* oocysts [22,42].

Regarding the effect of garlic extract and Allicin on the morphology of *Eimeria* oocysts, the highest deformity on oocysts was observed at concentrations of 90 mg/mL for garlic and 180 mg/mL for Allicin, with percentages of 24% (22/92) and 32.3% (45/139), respectively, while garlic contained 360 mg/mL. This result is consistent with a previous study [24], where allicin exhibits SH group reactivity on cysteine residues of pathogen enzymes. This activity resulted in the inactivation and inhibition of specific thiol-containing enzymes in microorganisms [43,44]. In this study, Allicin and alcoholic garlic extract-treated oocysts showed marked structural changes established by light and scanning microscope (SEM), which agrees with several previous reports [22,42] that estimated the effect of *Saccharum officinarum* extract and selenium-rich green tea extract on the sporulation of *Eimeria* oocysts. These studies were taken as references due to the lack of research papers on the effect of Allicin and alcoholic garlic extracts on the sporulation of *Eimeria* oocysts.

In our study, *Eimeria* oocysts sporulated in allicin exhibited the lowest pathogenicity compared to oocysts sporulated in media containing alcoholic garlic extract and KOH due to its direct effect on viability and, consequently, on the infectivity of the oocyst [2]. In contrast, *Eimeria* oocysts sporulated in allicin-containing media with the lowest in vivo gross lesion score and oocyst shedding compared to those sporulated in media containing alcoholic garlic extract and KOH. Regarding histopathological findings and scoring, birds challenged with non-treated oocysts exhibited hemorrhagic inflammatory lesions. Birds challenged with allicin-treated oocysts exhibited minimal histopathological changes, including necrosis at the tips of some villi, mononuclear cell infiltration in the lamina propria, and a small number of coccidian parasites in the villar epithelium. In light of the information mentioned above, this study describes the effect of Allicin and garlic on the viability and sporulation of oocysts and the infectivity of the different parasitic stages of *E. tenella*. Additional research is required to determine the efficacy of these plant extracts as disinfectants in poultry farms to control the spread of *E. tenella*.

## 5. Conclusions

This study concluded that Allicin and *Allium sativum* extracts possess anticoccidial properties against *E. tenella* in broiler chickens. As the in vitro application of these herbal products directly affected the number, viability, pathogenicity, and infectivity of *E. tenella*, it was determined that they were safe for human consumption. As a disinfectant, this could be a promising alternative to chemical compounds for controlling coccidiosis in poultry farms. Future research should focus on the mechanistic pathways underlying these natural substances against *E. tenella* stages and their potential activity against other parasites.

## Figures and Tables

**Table 1:** In vitro efficiency of different allicin and alcoholic garlic extract concentrations against *E. tenella* oocysts’ number and sporulation dynamic within 48 h post-exposure compared to KOH 5% (as a chemical disinfectant) *.

Compound	Conc. (mg/mL)	Oocysts Reduction %	Sporulation Dynamic	
			S%	SI%
Control (K_2_Cr_2_O_7_)	25	00.0 ± 0.0	51.2 ± 3.6 ^a^	00.0 ± 0.0
Allicin	45	56.6 ± 2.1 ^c^	27.4 ± 8.7 ^bc^	46.5 ± 1.4 ^b^
	90	72.7 ± 1.7 ^b^	26.1 ± 1.2 ^c^	49.0 ± 2.1 ^b^
	180	88.3 ± 1.2 ^a^	15.9 ± 0.9 ^d^	68.9 ± 2.1 ^a^
Garlic	90	26.8 ± 0.9 ^d^	28.2 ± 1.7 ^bc^	44.9 ± 3.6 ^b^
	180	38.4 ± 1.2 ^d^	27.2 ± 1.6 ^bc^	46.9 ± 1.0 ^b^
	360	73.5 ± 2.2 ^b^	13.6 ± 1.6 ^d^	73.4 ± 2.8 ^a^
KOH	50	67.4 ± 1.5 ^b^	35.6 ± 1.5 ^b^	30.5 ± 2.4 ^c^

* Values were represented in percentages. S%: Sporulation percent. SI%: Sporulation Inhibition percent. ^a–d^: values followed by different superscripts within a column are statistically different (*p* < 0.05; *p* < 0.01; *p* < 0.001) as indicated by ANOVA.

**Table 2:** Effect of different Allicin and alcoholic garlic extract concentrations compared to KOH 5% (as a chemical disinfectant) on the morphology of *Eimeria tenella* oocysts by light microscope and scanning electron microscope within 24 and 48 h post in vitro exposure.

Compound	Conc (mg/mL)	Oocysts Deformity %			
		24 h		48 h	
		Non-Sporulated	Sporulated	Non-Sporulated	Sporulated
Control (K_2_Cr_2_O_7_)	25	1	0	0	0
Allicin	45	35.1	0.90	32.3	21
	90	46.1	2.10	34.8	26.7
	180	51.9	5.70	41	32.3
Garlic	90	3.4	0.20	21.7	13.6
	180	6	2.40	28.3	22.8
	360	14.3	3.9	44	24
KOH	50	9.3	0.00	11.5	11.5

Data were recorded after light microscopy examination (40× and 100×). alues were expressed in percentage.

**Table 3:** Scoring of the histopathological findings of cecal sections of different experimental groups.

Lesions	Negative Control	Infected with Non-Treated Oocysts	Infected with Allicin-Treated Oocysts	Infected with Garlic Extract-Treated Oocysts	Infected with KOH-Treated Oocysts
Epithelial necrosis	0	5	1	3	4
Hemorrhages	0	5	0	0	0
Eosinophils infiltration	0	5	0	0	0
Mononuclear cells infiltration	0	5	2	3	3
Coccidial stages	0	5	1	3	3

(0, normal; 1, minimal severity; 2, mild severity; 3, moderate; 4, marked; and 5, severe).

## Data Availability

Not applicable.
